# Preliminary Evaluation of the Psychometric Properties of the Ukrainian Traumatic Grief Inventory-Self Report Plus (TGI-SR+)

**DOI:** 10.32872/cpe.17367

**Published:** 2026-05-29

**Authors:** Maria Luisa F. Rispa Hoyos, Lieke C. J. Nijborg, Iryna Norkina, Paul A. Boelen, Lonneke I. M. Lenferink

**Affiliations:** 1 Department of Psychology, Health & Technology, Faculty of Behavioural Management and Social Sciences, University of Twente, Enschede, The Netherlands; 2 Department of Clinical Psychology, Faculty of Social Sciences, Utrecht University, Utrecht, The Netherlands; 3 ARQ National Psychotrauma Centre, Diemen, The Netherlands; Philipps-University of Marburg, Marburg, Germany

**Keywords:** grief, war, loss, assessment, Russian invasion, bereavement

## Abstract

**Background:**

Prolonged Grief Disorder (PGD) was included in the DSM-5-TR and ICD-11. The Traumatic Grief Inventory Self-Report Plus (TGI-SR+) is a self-report measure to assess PGD symptoms in accordance with both classification systems. It has been translated into various languages and validated across different contexts.

**Objective:**

Evaluating the psychometric properties of the Ukrainian TGI-SR+.

**Method:**

Participants were Ukrainian adults who had lost a loved one at least 6 months ago. One hundred ninety-eight participants completed the TGI-SR+ and measures assessing posttraumatic stress and depression. We examined the factor structure, internal consistency, convergent validity, and known-groups validity of the TGI-SR+. Moreover, rates of probable PGD caseness were calculated, and provisional cut-off scores were determined based on Receiver Operating Characteristic (ROC) analyses.

**Results:**

The one-factor model showed an acceptable fit for DSM-5-TR, but not for ICD-11 PGD symptoms. While some of the factor loadings were low for both criteria sets, the items demonstrated good internal consistency. Convergent validity was supported by strong associations between symptom levels of DSM-5-TR and ICD-11 PGD and posttraumatic stress and depression severity scores. Known-groups validity was partially supported by DSM-5-TR and ICD-11 PGD severity being related to both cause of death and kinship to the deceased. The provisional cut-off score for detecting both probable DSM-5-TR and ICD-11 PGD caseness, when summing all TGI-SR + items, was ≥ 75.

**Conclusion:**

The psychometric properties of the Ukrainian TGI-SR+ were mixed. However, pending further research in different and larger samples of Ukrainian bereaved people, this instrument can be used to assess PGD severity in Ukrainians.

## Highlights

- Prolonged grief symptoms were prevalent among Ukrainians and strongly correlated with posttraumatic stress and depressive symptoms.
- Losses due to violent or unexpected causes were associated with more severe and persistent prolonged grief symptoms than natural losses.
- Provisional cut-off scores were identified to screen for probably prolonged grief disorder among bereaved Ukrainians.

The death of a loved one is a potentially stressful life event that can have a substantial negative effect on the mental health of those left behind (Seiler et al., 2020). Most individuals can adjust to the loss (Nielsen et al., 2019), but about 3-5% experience persistent grief symptoms after losing someone to a natural cause (Rosner et al., 2021; Treml, Linde, et al., 2024), and this rate is four times higher in cases of unexpected losses (e.g., accidents) (Doering et al., 2022).

The definition of pathological grief has generated discussion among experts (Eisma, 2023; Eisma et al., 2022; Haneveld et al., 2022; Lenferink et al., 2021; Prigerson et al., 2024). The fifth edition of the Diagnostic and Statistical Manual of Mental Disorders (DSM-5) included Persistent Complex Bereavement Disorder (PCBD) (American Psychiatric Association [APA], 2013) and was replaced by Prolonged Grief Disorder (PGD) in the text revision of the DSM-5 (i.e., DSM-5-TR; APA, 2022). According to the DSM-5-TR, PGD encompasses two separation distress symptoms (i.e., intense yearning or longing for, and preoccupation with thoughts or memories of, the deceased person), and eight accompanying symptoms (e.g., identity disruption, intense emotional pain, and emotional numbness; APA, 2022). DSM-5-TR PGD can be diagnosed one year after the loss for adults and six months for children, when the bereaved person experiences at least one separation distress symptom and three or more accompanying symptoms daily for at least a month, exceeding social, cultural, or religious norms and causing significant distress or impairment in daily activities.

PGD has also been included in the 11th edition of the International Classification of Diseases (ICD-11; World Health Organization [WHO], 2025). Diagnostic criteria in ICD-11 differ slightly from those in DSM-5-TR in terms of the number and content of symptoms. ICD-11 PGD has two separation distress symptoms and ten accompanying symptoms (e.g., sadness, guilt, anger, and denial) and can be diagnosed six months after the loss for both adults and children, when the bereaved person experiences at least one separation distress symptom and one accompanying symptom daily for at least a month, causing functional impairment.

Several instruments have been created to assess PGD levels according to the DSM-5-TR (e.g., Prolonged Grief Disorder-13 – Revised, Prigerson et al., 2021) and ICD-11 diagnostic criteria sets (e.g., International Prolonged Grief Disorder Scale, Killikelly et al., 2020), separately. However, assessing DSM-5-TR and ICD-11 PGD symptoms simultaneously using one measurement tool, such as the Traumatic Grief Inventory-Self Report Plus (TGI-SR+; Lenferink et al., 2022), has several advantages. The TGI-SR+ has been translated into more than 15 different languages and is freely accessible on the Open Science Framework, facilitating cross-national assessment of PGD severity (see https://osf.io/rqn5k/), while mitigating barriers to comparing results across cultures (Comtesse et al., 2024).

The Dutch TGI-SR+ has been validated in distinct samples of people bereaved by various causes and people bereaved by traffic accidents. In both samples, the TGISR+ demonstrated robust psychometric properties, as indicated by good construct validity, internal consistency, temporal stability, convergent and known-groups validity (Lenferink et al., 2022). These findings have been replicated with the French TGI-SR+ with young adults (Kokou-Kpolou et al., 2022), the Swedish TGI-SR+ in bereaved parents (Lenferink, Van Dijk, et al., 2024), the Norwegian TGI-SR+ in people whose child or sibling died unexpectedly or violently (Lenferink, Johnsen, et al., 2024), and the Chinese TGI-SR+ in adults bereaved due to COVID-19 (Tang et al., 2024). However, validation remains largely concentrated in Western Europe. Expanding the psychometric evaluation to non-Western countries is critical, particularly in regions with high mortality rates and armed conflicts, where the risk for PGD is elevated (Kokou-Kpolou et al., 2020).

A reliable and valid screening tool for PGD is crucial for identifying people who may need professional support. The present study evaluated the psychometric properties of the Ukrainian TGI-SR+ to assess DSM-5-TR and ICD-11 based PGD symptoms. The Russian invasion resulted in one of Europe's largest forced displacement crises, affecting more than 44 million Ukrainians (Shevlin et al., 2022), with over 100,000 refugees in the Netherlands (Government of the Netherlands, 2024). Forced refugees have a high risk of developing mental health conditions, such as posttraumatic stress disorder (PTSD), depression, and anxiety (Charlson et al., 2019; Steel et al., 2009). PGD has also been observed in displaced populations (Bryant et al., 2020) and may be commonplace among Ukrainians (Killikelly et al., 2024; Shevlin et al., 2022).

To psychometrically evaluate the Ukrainian TGI-SR+, we evaluated the factor structure of both DSM-5-TR PGD and ICD-11 PGD. Based on prior research (Kokou-Kpolou et al., 2022; Lenferink et al., 2022; Lenferink, Johnsen, et al., 2024), we expected the one-factor model to fit the data best for both DSM-5-TR PGD and ICD-11 PGD. Moreover, we expected to find high internal consistency (Kokou-Kpolou et al., 2022; Lenferink et al., 2022; Lenferink, Van Dijk, et al., 2024; Tang et al., 2024).

To demonstrate convergent validity, we examined to what extent DSM-5-TR PGD and ICD-11 PGD levels are associated with PTSD and depression levels, expecting to find strong positive correlations between these constructs (cf. Boelen, 2021; Eisma et al., 2019; Kokou-Kpolou, 2021; Lenferink et al., 2022; Maccallum & Bryant, 2018; Schaal et al., 2012). Regarding known-groups validity, we hypothesized that people self-identified as women (vs. men) and those who lost a parent, partner, or child (vs. a more distant close person) would report higher DSM-5-TR and ICD-11 PGD levels. Also, we expected that losses due to an unnatural cause (e.g., homicide, accident, suicide) would be associated with higher DSM-5-TR and ICD-11 PGD levels than losses due to a natural cause (e.g., old age, chronic illness) and that time since loss would be negatively associated with these PGD levels (Buur et al., 2024; Doering et al., 2022). Lastly, we determined cut-off scores to distinguish between people who meet the criteria for probable DSM-5-TR PGD and ICD-11 PGD caseness and those who do not.

## Material and Method

### Participants and Procedures

Data were collected online from Ukrainian adults (≥ 18 years) who had lost a loved one at least six months earlier (for recruitment strategies and procedures, see Supplementary Material A).

In total, 430 individuals started the survey. We excluded responses from people whose loved one had died less than six months before completing the survey (*n* = 140), who did not complete all three main instruments (TGI-SR+, PCL-5, and PHQ-9; *n* = 81), who were minors, duplicate entries, or test responses (*n* = 7), and who provided ambiguous answers regarding the cause of death (e.g., going different ways; *n* = 4). The final sample included 198 participants.

### Measures

#### Sociodemographic and Loss-Related Characteristics

The following sociodemographic and loss-related characteristics were assessed: gender (1 = man, 2 = woman, 3 = other), age (in years), whether the participant fled their home due to war (1 = no, 2 = yes, I fled my house and live somewhere else in Ukraine, and 3 = yes, now I live in a different country), date of death of their deceased loved one, cause of death (1 = physical illness, 2 = accident, 3 = suicide, 4 = murder or manslaughter not related to war, 5 = killed in action/combat, 6 = disappearance, 7 = other), and kinship (1 = partner, 2 = child, 3 = parent, 4 = sibling, 5 = grandparent, 6 = grandchild, 7 = friend, and 8 = other).

#### Traumatic Grief Inventory-Self-Report Plus (TGI-SR+)

The TGI-SR+ is a 22-item extended version of the original TGI-SR (Boelen & Smid, 2017) designed to assess PGD severity as defined in the DSM-5-TR and ICD-11 (Lenferink et al., 2022). Participants rated each item from 1 (never) to 5 (always) to indicate the extent to which they experienced a symptom during the past month (e.g., I avoided places, objects, or thoughts that reminded me that the person I have lost has died). For information about the translation, see Supplementary Material B.

#### Posttraumatic Stress Disorder Checklist (PCL-5) 6-Item Short Form

PTSD severity was assessed using a six-item ICD-11 subset of the PCL-5 (Items 2, 3, 6, 7, 17, and 18; Heeke et al., 2022; Weathers et al., 2013). Participants rated symptom presence in the preceding month on a 5-point scale with anchors 1 (not at all) and 5 (extremely), with total scores ranging from 6 to 30. The scores were dichotomized such that Scores 1 – 2 indicated symptom absence, while scores ≥ 3 denoted symptom presence (Heeke et al., 2022). Probable caseness required the endorsement of all items.

The 6-item PCL-5 has good psychometric properties (Heeke et al., 2022). The wording of items was changed to refer to the loss instead of the stressful event (e.g., In the past month, how much were you bothered by avoiding external reminders of the death of your loved one (for example, people, places, conversations, activities, objects, or situations)?). We used the items from the Ukrainian version of PCL-5, which has satisfactory psychometric properties (Johnson et al., 2022; Roberts et al., 2019; Ukrainian translation: International Trauma Consortium, n.d.). In this present study, the 6-item PCL-5 showed acceptable internal consistency (ω = .76).

#### Patient Health Questionnaire – 9 (PHQ-9)

The PHQ-9 consists of nine items used to assess depression severity in the last two weeks (e.g., feeling down, depressed, or hopeless; Kroenke et al., 2001). Each item was rated from 0 (not at all) to 3 (almost every day). A total depression score was calculated by summing all items (range: 0–27). A total score ≥ 10 indicates probable caseness of depression (Gilbody et al., 2007; Kroenke et al., 2001). For this study, the validated Ukrainian translation of the PHQ-9 was used (Hyland et al., 2023; Riad et al., 2022; Ukrainian translation: International Trauma Consortium, n.d.). Internal consistency in the present sample was good (ω = .86).

### Statistical Analysis

Confirmatory factor analyses (CFAs) were performed using Mplus 7.4 (Muthén & Muthén, 1998). The remaining analyses were performed using IBM SPSS, version 28.0 (IBM Corporation, 2017).

#### Factor Structure of DSM-5-TR and ICD-11-Based PGD Symptoms

Kurtosis and skewness values were examined. Kurtosis values < 10 and skewness values < 3 are indicative of a univariate normal distribution of item scores (Kline, 2011). All kurtosis and skewness values were < 2; consequently, the default maximum likelihood estimator was used. There were no missing data on PGD items.

CFAs were performed to evaluate the factor structure of DSM-5-TR PGD and ICD-11 based PGD symptoms. First, a one-factor model was evaluated for DSM-5-TR PGD and ICD-11 PGD, separately. Second, following prior research (Kokou-Kpolou et al., 2022; Lenferink et al., 2022; Lenferink, Johnsen, et al., 2024; Lenferink, Van Dijk, et al., 2024; Tang et al., 2024), a two-factor model was evaluated. In the two-factor model, the two items representing separation distress (i.e., the yearning and preoccupation items) load on one factor, while the items representing accessory symptoms load on a second factor.

Model fit was evaluated using the Comparative Fit Index (CFI) and Tucker-Lewis Index (TLI), where values > .95 indicate excellent fit and > .90 acceptable fit. The root-mean-square error of approximation (RMSEA) and standardized root mean square residual (SRMR) were also evaluated, with values < .05 indicating excellent fit and < .10 indicating acceptable fit (Kline, 2011). In addition, the lower the Akaike information criterion (AIC) and Bayesian information criterion (BIC), the better the fit. Factor loadings below 0.6 are considered low.

#### Internal Consistency

Reliability of the DSM-5-TR PGD and ICD-11 PGD items was evaluated using McDonald’s Omega (ω). Values of ω > 0.70 indicate acceptable internal consistency (Hayes & Coutts, 2020; Trizano-Hermosilla & Alvarado, 2016).

#### Convergent Validity

To examine the convergent validity of the TGI-SR+, correlation analyses were performed. Based on Kolmogorov-Smirnov normality tests, summed scores on the items representing the DSM-5-TR PGD and ICD-11 PGD symptoms were non-normally distributed (i.e., *p* < .05). Spearman’s Rho correlations were calculated to examine associations between DSM-5-TR PGD and ICD-11 PGD total scores and PTSD and depression total scores. Correlations ≤ 0.29 were considered weak, between 0.30 and 0.49 moderate, and ≥ 0.50 strong (Cohen, 1988).

#### Known-Groups Validity

Mann-Whitney U tests were carried out to examine whether DSM-5-TR PGD and ICD-11 PGD total scores differed as a function of gender cause of death (dichotomized into unnatural cause of death (i.e., accident, suicide, killed in combat, disappearance) and natural cause of death [i.e., physical illness, old age, died at birth, etc.]), and kinship (dichotomized into nuclear family member [i.e., child, partner, parent| and other).

Moreover, it was examined whether the time since loss (in months) was related to PGD total scores, using Spearman’s Rho correlations.

#### Rate of Probable PGD Caseness Using Diagnostic Scoring Rules

A participant qualified for probable DSM-5-TR PGD caseness when endorsing at least one of the two Criterion B (i.e., separation distress) symptoms (Items 1 and 3), three or more of the eight Criterion C (i.e., cognitive, emotional, and behavioral) symptoms (Items 6, 9, 10, 11, 19, 21, and the highest score indicated on items 2 and 8^1^1One symptom, emotional pain, of criterion C (i.e., C4) is represented by two items (2 and 8).), and item 13, representing Criterion D (i.e., functional impairment; APA, 2022).

A participant qualified for probable ICD-11 PGD caseness when endorsing at least one of the two criterion B symptoms (Items 1 and 3), at least one of the 10 criterion C symptoms (Items 2, 5, 8, 9, 10, 16, 19, 20, 21, and 22), and item 13, representing Criterion D (i.e., functional impairment; World Health Organization, 2025). Items were considered “endorsed” when scored at least 3.

#### Optimal Cut-Off Scores

By performing receiver operating characteristic (ROC) analyses, we determined the optimal cut-off scores to identify probable DSM-5-TR and ICD-11 PGD cases. Specifically, we examined optimal cut-off scores based on (1) the total score of all 22 TGI-SR+ items (range: 22-110), (2) the total score of all DSM-5-TR based PGD items, excluding the functional impairment item (range: 10-50), and (3) the total score of all ICD-11 based PGD items, excluding the functional impairment item (range: 12–60).

A ROC was plotted with the true positive ratio (i.e., sensitivity) as a function of the false positive ratio (i.e., 1 – specificity) for each possible total score. Area Under the Curve (AUC) values ​​≥ 0.90 indicate that the score has excellent accuracy in distinguishing probable “cases” from “non-cases” (Ferraris, 2019). In addition, the Youden index (i.e., sensitivity index – [1 – specificity index]) was calculated. Values between 0.90 and 1 are excellent and indicate high diagnostic accuracy (Schisterman et al., 2005), between 0.80 and 0.90 are considered good, and between 0.70 and 0.80 are fair. When values < .70, this indicates a poor accuracy in distinguishing probable caseness from probable non-caseness (Ferraris, 2019).

## Results

### Sample Characteristics

The characteristics of the sample are shown in Table 1. Nine out of 10 participants self-identified as women. On average, participants were 36 years old (*SD* = 8.79). Approximately three out of 10 participants fled from Ukraine to another country due to the war with Russia. Most participants lost their loved one due to a physical illness, followed by being killed in action/combat, or an accident. A majority of participants reported that they had lost a nuclear family member. On average, the loss took place around four years ago. About one out of 10 people met the criteria for probable PTSD, and six out of 10 people met the criteria for probable depression.

**Table 1:** Characteristics of the Sample (N = 198)

Variable	*n* (%) or *M* (*SD*) & range
Gender (*n*, %)	
Male	7 (3.5)
Female	184 (92.9)
Other	7 (3.5)
**Age (in years; *M*, *SD*, range)**	36.23 (8.79); 19 – 59
Fled home due to war (*n*, *%*)	
No	111 (56.1)
Yes, inside Ukraine	28 (14.1)
Yes, to another country	59 (29.8)
Cause of death (*n*, *%*)	
Physical illness (e.g., cancer, cardiovascular disease, died at birth)	116 (58.6)
Accident (e.g., traffic accident, drowning, poisoning)	19 (9.6)
Suicide	10 (5.1)
Murder or manslaughter not related to war	3 (1.5)
Killed in action/combat	37 (18.7)
Disappearance	4 (2.0)
Other	9 (4.5)
Kinship, the deceased was the participant’s … (*n*, *%*)	
Partner (husband, wife, boyfriend, girlfriend)	42 (21.2)
Child	26 (13.1)
Parent	73 (36.9)
Sibling	17 (8.6)
Grandparent	18 (9.1)
Friend(s)	12 (6.1)
Other	10 (5.1)
**Time since the loss (in months; *M*, *SD*, range)**	45.85 (58.50); 6 – 422
Psychological Outcomes (*M*, *SD*, range)	
DSM-5-TR PGD levels (TGI-SR+)	32.48 (7.85); 13 – 49
ICD-11 PGD levels (TGI-SR+)	40.12 (8.67); 15 – 58
PTSD levels (PCL-5)	14.27 (4.77); 6 – 26
Depression levels (PHQ-9)	11.30 (6.09); 0 – 25

*Note.* DSM-5-TR = 5th text revised edition of the Diagnostic and Statistical Manual of Mental Disorders; ICD-11 = 11th edition of the International Classification of Diseases; PCL-5 = Posttraumatic stress disorder Checklist for DSM-5; PGD = Prolonged Grief Disorder; PHQ-9 = Patient Health Questionnaire 9; PTSD = posttraumatic stress disorder; TGI-SR + = Traumatic Grief Inventory – Self Report Plus.

### Factor Structure of DSM-5-TR and ICD-11 PGD Items

For DSM-5-TR PGD, both the one- and two-factor models showed acceptable fit, as indicated by the CFI, TLI, RMSEA, and SRMR values (see Table 2). However, the two-factor model did not fit significantly better than the one-factor model (Δχ^2^ (Δ*df*) = 2.36 (1), *p* < .20) and, accordingly, the one-factor and two-factor models had similar AIC and BIC values. The one-factor model was therefore selected as the optimal model (see Table 3 for the standardized factor loadings), despite very low factor loadings for the items “I had intrusive thoughts or images related to the person who died” and “I avoided places, objects, or thoughts that reminded me that the person I lost has died”.

**Table 2:** Fit Indices of the Confirmatory Factor Analyses (N = 198)

Model	χ^2^	*df*	CFI	TLI	RMSEA [90% CI]	SRMR	AIC	BIC
DSM-5-TR PGD								
One-factor	92.36	35	.93	.91	.09 [.07, .11]	.05	5538.97	5637.62
Two-factor	90.00	34	.93	.91	.09 [.07, .11]	.05	5538.61	5640.54
ICD – 11 PGD								
One-factor	169.06	54	.87	.84	.10 [.09, .12]	.06	6680.38	6798.76
Two-factor	166.67	53	.87	.84	.10 [.09, .12]	.06	6679.99	6801.65

*Note.* AIC = Akaike Information Criterion; BIC = Bayesian Information Criterion; CFI = Comparative Fit Index; CI = Confidence Interval; DSM-5-TR = Diagnostic and Statistical Manual of Mental Disorders 5 Text-Revision; ICD-11 = the 11th edition of the International Classification of Diseases; PGD = Prolonged Grief Disorder; RMSEA = Root Mean Square Error of Approximation; SRMR = Standardized Root Mean Square Residual; TLI = Tucker Lewis Index.

**Table 3:** Standardized Factor Loadings One-Factor Model for DSM-5-TR and ICD-11 PGD (N = 198)

Variable	Est.	*SE*
1 factor DSM-5-TR PGD		
I found myself longing or yearning for the person who died.	.596	.050
I had intrusive thoughts or images related to the person who died.	.123	.073
It felt as if a part of me has died along with the deceased	.776	.032
It felt unreal that he/she is dead	.558	.053
I avoided places, objects, or thoughts that reminded me that the person I lost has died	.229	.068
I experienced intense emotional pain, sadness, or pangs of grief/I felt bitterness or anger related to his/her death.	.670	.043
I felt that that moving on (e.g., making new friends, pursuing new interests) was difficult for me.	.765	.034
I felt emotionally numb.	.726	.038
I felt that life is unfulfilling or meaningless without him/her.	.880	.022
I felt alone or detached from other individuals.	.725	.038
1 factor ICD-11 PGD		
I found myself longing or yearning for the person who died.	.677	.044
I had intrusive thoughts or images related to the person who died.	.096	.075
I experienced intense emotional pain, sadness, or pangs of grief.	.760	.035
I had negative thoughts about myself in relation to the loss (e.g., thoughts about self-blame).	.472	.060
I felt bitterness or anger related to his/her death.	.533	.055
It felt unreal that he/she is dead	.621	.048
I put an intense blame on others because of his/her death	.305	.069
I had trouble accepting the loss.	.709	.041
It felt as if a part of me has died along with the deceased	.741	.037
I had difficulties experiencing positive feelings	.698	.042
I felt emotionally numb.	.687	.043
I felt that moving on (e.g., making new friends, pursuing new interests) was difficult for me.	.697	.042

*Note.* DSM-5-TR = Diagnostic and Statistical Manual of Mental Disorders 5 Text-Revision; ICD-11 = the 11th edition of the International Classification of Diseases; PGD = Prolonged Grief Disorder.

For ICD-11 PGD, none of the fit indices indicated an acceptable fit for the one-factor or the two-factor model, except for the SRMR values (see Table 2). When comparing the fit of the two-factor model to the one-factor model, there was no significant improvement in fit (Δχ^2^ (Δ*df*) = 2.39 (1), *p* < .20), and AIC and BIC values of both models were similar. Table 3 shows the factor loadings of the one-factor model. Again, some of the factor loadings were very low, with the lowest loadings for the following two items: “I had intrusive thoughts or images related to the person who died” and “I put an intense blame on others because of his/her death”.

### Internal Consistency

The DSM-5-TR PGD items and the ICD-11 PGD items displayed acceptable internal consistency (both ω = .86).

### Convergent Validity

As expected, significant, positive, and strong associations were found between DSM-5-TR PGD levels and PTSD (ρ  =  .54, *p*  <  .001) and depression levels (ρ  =  .50, *p*  <  .001). Significant, positive, and strong associations were also found between ICD-11 PGD levels and PTSD (ρ  =  .53, *p*  <  .001) and depression levels (ρ  =  .51, *p*  <  .001).

### Known-Groups Validity

As expected, participants who lost a nuclear family member reported more severe DSM-5-TR PGD symptoms than those who lost another loved one (*U* = 3293.5, *p* = .047). Kinship was unrelated to ICD-11 PGD severity (*U* = 3383.0, *p* = .081). Moreover, participants who lost someone due to an unnatural cause reported higher symptoms of DSM-5-TR PGD (*U* = 3072.5, *p* <.001) and ICD-11 PGD (*U* = 3057.5, *p* <.001) than those who lost someone due to a natural cause.

Time since loss was significantly and negatively associated with the severity of DSM-5-TR PGD (ρ = -.162, *p* = .02) and trended toward being significantly associated with ICD-11 PGD severity (ρ = -.133, *p* = .06). Due to the overrepresentation of women, we were unable to examine gender differences.

### Rates of Probable PGD Caseness

Seventy-five participants (37.9%) met criteria for probable DSM-5-TR PGD caseness, whereas 80 (40.4%) met criteria for probable ICD-11 PGD caseness.

### Optimal Cut-Off Scores

When summing all 22-item TGI-SR+, the optimal cut-off score indicative of probable DSM-5-TR PGD caseness was ≥ 75 (AUC = 0.921, 95% CI: 0.884–0.957). The Youden's index suggested poor diagnostic accuracy (J = 0.65). With this cut-off score, 78% of probable cases were correctly identified as DSM-5-TR PGD cases, and 13% were incorrectly identified. When summing the 10 DSM-5-TR PGD items (range between 10-50), the optimal cut-off score indicative of probable DSM-5-TR PGD caseness was ≥ 34 (AUC = 0.919, 95% CI: 0.882–0.955). With this cut-off score, 85% of probable cases were correctly identified as a DSM-5-TR PGD case, and 17% were incorrectly identified. The Youden's index suggested poor diagnostic accuracy (J = 0.68).

For ICD-11 PGD, when summing all 22 items, the optimal cut-off score was also ≥ 75 (AUC = 0.905, 95% CI: 0.865–0.945). The Youden's index was fair (J = 0.71) and 83% of probable cases were correctly identified as ICD-11 PGD cases, whereas 12% were incorrectly identified. When summing the 12 ICD-11 PGD items (range 12-60), the optimal cut-off score was ≥ 44 (AUC = 0.875, 95% CI: 0.826–0.924); 76% of probable cases were correctly identified as an ICD-11 case, and 15% were incorrectly identified. The Youden's index again suggested poor diagnostic accuracy (J = 0.61)

## Discussion

This cross-sectional study evaluated the psychometric properties of the Ukrainian TGI-SR+ to assess PGD according to the DSM-5-TR and ICD-11, among 198 bereaved Ukrainians.

We assessed the factor structure according to both the DSM-5-TR and ICD-11 diagnostic criteria set. We found that, as expected, the one-factor model showed adequate fit for the DSM-5-TR PGD items. A poor model fit was found for the ICD-11 PGD items. Notably, for both one-factor DSM-5-TR and ICD-11 PGD models, we found that some items had low factor loadings (e.g., Items 6, 8, 16, and 19, 20), indicating weak inter-item correlations. More specifically, Item 1, “I had intrusive thoughts or images related to the person who died”, had the lowest factor loading in both models. This result stands in contrast to prior research, which had shown that this item has strong factor loadings. (Cherblanc et al., 2026; Lenferink, Johnsen, et al., 2024; Treml, Schmidt, et al., 2024). One explanation could be related to the challenge of translating the content of the item into Ukrainian with a clear and unambiguous meaning, which is a common challenge in cross-cultural studies (Cruchinho et al., 2024). These items should be re-translated and reassessed in future research. In addition, the reductionist and stigmatizing culture of how mental health is often viewed among Ukrainians (Frankova et al., 2024) may also play a role in the interpretation of the items by participants and the potential incomprehensibility of the terminology used.

Concerning the poor model fit for the ICD-11 PGD, the items reflecting self-blame and blaming others showed relatively low factor loadings, mirroring previous findings (see Lenferink, Johnsen, et al., 2024; Lenferink, Van Dijk, et al., 2024). This suggests that these items may not align well with the core symptoms of PGD and may contribute to the poor factor structure and overall fit of the ICD-11 model in this study.

Notably, we found acceptable internal consistency for both the DSM-5-TR PGD and ICD-11 PGD items, consistent with previous research (Lenferink et al., 2022; Lenferink, Van Dijk, et al., 2024). Furthermore, the convergent validity was further evidenced by strong positive correlations with measures of PTSD and depression, aligning with the literature (Eisma et al., 2019; Fernández-Alcántara et al., 2025; Kokou-Kpolou, 2021; Kokou-Kpolou et al., 2022; Lenferink, Johnsen, et al., 2024).

Regarding known-groups validity, individuals who lost a close relative or experienced a more recent loss reported higher DSM-5-TR, but not ICD-11, PGD scores. In addition, those who experienced an unnatural loss (e.g., due to an accident) reported higher DSM-5-TR and ICD-11 PGD levels than those who experienced a natural loss. These findings accord with prior evidence that a close relationship to the deceased, a more recent loss, and the unnatural death of a loved one are risk factors for PGD (Buur et al., 2024). There was no significant association between ICD-11 PGD and kinship nor time since loss, which might be related to the poor factor structure of the ICD-11 PGD items.

The association between time since loss and PGD severity was almost similar for DSM-5-TR and ICD-11, with the latter showing a marginally significant effect (Pritschet et al., 2016), suggesting that the lack of significance may be due to limited statistical power. This could explain why we did find significant differences in ICD-11 PGD levels regarding kinship.

The cut-off scores for probable PGD were ≥ 34 for DSM-5-TR PGD (when summing the 10 items) and ≥ 44 for ICD-11 PGD (when summing the 12 items). These cut-off scores are similar to those found in prior research among Dutch, Chinese, German, and Swedish bereaved people, which ranged from 32 to 34 and from 39 to 44, respectively (Lenferink et al., 2022; Lenferink, Van Dijk, et al., 2024; Tang et al., 2024; Treml, Schmidt, et al., 2024). When summing all 22 TGI-SR+ items, the optimal cut-off score for determining probable DSM-5-TR and ICD-11 PGD caseness was ≥ 75. These findings also align with the cut-off scores found in prior research, which varied from 65 to 71 for DSM-5-TR PGD and from 60 to 75 for ICD-11 PGD (Lenferink, Johnsen, et al., 2024; Lenferink, Van Dijk, et al., 2024). These differences in cut-off scores across studies may partly be explained by differences in the accuracy of determining them. In our sample, we obtained poor Youden's indices, indicating low precision in differentiating between bereaved people with and without probable PGD, which is likely due to the combination of our relatively small sample and low factor loadings for some of the items.

When interpreting the results, it is important to consider several limitations. First, the sample was predominantly female; while this is common among bereavement-related research (Eisma & Stroebe, 2021; Kokou-Kpolou et al., 2022; Lenferink et al., 2022), this overrepresentation prevented gender-based comparisons. Notably, in this particular case, the lack of men participating could reflect the ongoing Russian invasion of Ukraine, as data collection coincided with military mobilization. Related to this, some participants recently fled their country or may have been exposed to war-related stressors, which may have affected the results. The underrepresentation of men may also stem from cultural factors, Ukrainian men are generally (even) less open about their emotional state than men from Western countries (Plan International, 2025).

Second, due to the cross-sectional design of the present study, it was impossible to assess the test-retest reliability and predictive validity of the TGI-SR+. Longitudinal research is necessary to evaluate these crucial psychometric properties. Third, while the TGI-SR+ is a valuable tool to assess PGD severity, it is essential to corroborate self-reported symptoms with clinician-administered diagnostic interviews. Future research should employ validated clinical interviews to accurately assess the prevalence of PGD and more reliable cut-off scores. In the present study, ROC analyses were conducted using the self-report data, as no independent diagnostic interviews were available at the time of data collection. While this is common practice in studies evaluating assessment instruments for PGD (Fernández-Alcántara et al., 2025; Kokou-Kpolou et al., 2020; Lenferink, Johnsen, et al., 2024; Lenferink, Van Dijk, et al., 2024; Treml, Schmidt, et al., 2024), the cut-off scores derived from these analyses should be considered provisional, intended to provide clinicians with a rapid impression of potential PGD caseness, and should never replace a thorough clinical evaluation.

Additionally, while this study examined convergent validity by correlating PGD scores with PTSD and depression, divergent validity was not assessed. Although our CFA suggested a potential two-factor pattern for PGD items, the construct validity of each factor was not formally evaluated. Given the high correlation between the two factors, we did not expect significant differences in their construct validity. Therefore, we did not conduct a separate evaluation of the construct validity for each factor.

To conclude, we found mixed results regarding the psychometric properties of the Ukrainian TGI-SR+. The poor factor loadings of some items need further examination to improve the scale’s validity. Despite this, the instrument offers the possibility to assess PGD severity, a priority given the psychological impact of the ongoing war.

## Supplementary Materials

The Supplementary Materials contain the following items:

- **Research data and codebook** (Rispa Hoyos & Lenferink, 2026S)
- **Additional information** (Rispa Hoyos et al., 2026S):
  - *Supplementary Material A: Recruitment Strategies and Procedures.* Description of the recruitment strategies and procedures used in the study.
  - *Supplementary Material B: TGI-SR+ translation.* Description of the manner in which the TGI-SR+ was translated.

Rispa HoyosM. L. F.
LenferinkL. I. M.
 (2026S). Validation of the Ukranian Traumatic Inventory Self-Report (TGISR+)"
[Research data and codebook]. PsychOpen. 10.17026/SS/OVYGII


Rispa HoyosM. L. F.
NijborgL. C. J.
NorkinaI.
BoelenP. A.
LenferinkL. I. M.
 (2026S). Supplementary materials to "Preliminary evaluation of the psychometric properties of the Ukrainian Traumatic Grief Inventory-Self Report Plus (TGI-SR+)"
[Additional information]. PsychOpen. 10.23668/psycharchives.21853


## Data Availability

The data that support the findings of this study are available in the Data Archiving and Networked Services (DANS) repository at https://doi.org/10.17026/SS/OVYGII

## Preregistration

This study was not preregistered.

## Social Media Accounts

*Maria Luisa F. Rispa Hoyos:* LinkedIn, *Lieke C. J. Nijborg:* LinkedIn, *Iryna Norkina:* LinkedIn, *Paul A. Boelen:* LinkedIn, *Lonneke I. M. Lenferink:* LinkedIn

## Reporting Guidelines

This article was prepared following the JARS-Quant guidelines.

## References

[sp1_r1] Rispa Hoyos M. L. F. Lenferink L. I. M. (2026 S). Validation of the Ukranian Traumatic Inventory Self-Report (TGISR+)" [Research data and codebook]. PsychOpen. 10.17026/SS/OVYGII

[sp1_r2] Rispa Hoyos M. L. F. Nijborg L. C. J. Norkina I. Boelen P. A. Lenferink L. I. M. (2026 S). Supplementary materials to "Preliminary evaluation of the psychometric properties of the Ukrainian Traumatic Grief Inventory-Self Report Plus (TGI-SR+)" [Additional information]. PsychOpen. 10.23668/psycharchives.21853

